# Effects and Mechanisms of Non-Thermal Plasma-Mediated ROS and Its Applications in Animal Husbandry and Biomedicine

**DOI:** 10.3390/ijms242115889

**Published:** 2023-11-02

**Authors:** Yuhan Yang, Yuan Wang, Shang Wei, Xianzhong Wang, Jiaojiao Zhang

**Affiliations:** Chongqing Key Laboratory of Forage and Herbivore, College of Veterinary Medicine, Southwest University, Chongqing 400715, China; y18696765428@163.com (Y.Y.); w8914112023@163.com (Y.W.); w18680704934@163.com (S.W.); wang1973@swu.edu.cn (X.W.)

**Keywords:** non-thermal plasma, reactive oxygen species, animal husbandry, biomedicine

## Abstract

Non-thermal plasma (NTP) is an ionized gas composed of neutral and charged reactive species, electric fields, and ultraviolet radiation. NTP presents a relatively low discharge temperature because it is characterized by the fact that the temperature values of ions and neutral particles are much lower than that of electrons. Reactive species (atoms, radicals, ions, electrons) are produced in NTP and delivered to biological objects induce a set of biochemical processes in cells or tissues. NTP can mediate reactive oxygen species (ROS) levels in an intensity- and time-dependent manner. ROS homeostasis plays an important role in animal health. Relatively low or physiological levels of ROS mediated by NTP promote cell proliferation and differentiation, while high or excessive levels of ROS mediated by NTP cause oxidative stress damage and even cell death. NTP treatment under appropriate conditions not only produces moderate levels of exogenous ROS directly and stimulates intracellular ROS generation, but also can regulate intracellular ROS levels indirectly, which affect the redox state in different cells and tissues of animals. However, the treatment condition of NTP need to be optimized and the potential mechanism of NTP-mediated ROS in different biological targets is still unclear. Over the past ten decades, interest in the application of NTP technology in biology and medical sciences has been rapidly growing. There is significant optimism that NTP can be developed for a wide range of applications such as wound healing, oral treatment, cancer therapy, and biomedical materials because of its safety, non-toxicity, and high efficiency. Moreover, the combined application of NTP with other methods is currently a hot research topic because of more effective effects on sterilization and anti-cancer abilities. Interestingly, NTP technology has presented great application potential in the animal husbandry field in recent years. However, the wide applications of NTP are related to different and complicated mechanisms, and whether NTP-mediated ROS play a critical role in its application need to be clarified. Therefore, this review mainly summarizes the effects of ROS on animal health, the mechanisms of NTP-mediated ROS levels through antioxidant clearance and ROS generation, and the potential applications of NTP-mediated ROS in animal growth and breeding, animal health, animal-derived food safety, and biomedical fields including would healing, oral treatment, cancer therapy, and biomaterials. This will provide a theoretical basis for promoting the healthy development of animal husbandry and the prevention and treatment of diseases in both animals and human beings.

## 1. Introduction

Physical plasmas are exited ionized gases, which contain different concentrations of charged particles (electrons, positive and negative ions), radicals, neutral species (atoms and molecules), photons (visible and ultraviolet (UV) light), and electromagnetic fields that are generated by energy supply to a neutral gas [1]. Among the many distinctions between different kinds of plasmas, one is between thermal and non-thermal plasmas. Within thermal plasma, the electrons and the ions have the same energy. Non-thermal plasma (NTP) obtains its reactivity from the high energy electrons, while the ions and neutral species remain cold, which is suitable for life science researches and applications [2]. NTP has a very high chemical activity, and it can be ionized under the action of an applied electric field to produce a large number of electrons, ions, and atoms in the ground state; numerous molecules, atoms, and free radicals in the excited state; as well as rays and other mixtures [3]. There are two main types of NTP devices: direct NTP and indirect NTP. Direct NTP involves one of the electrodes being part of the electrical circuit of the treated object, it includes corona discharge, dielectric barrier discharge (DBD) volume, and spark discharge; indirect NTP treatment involves the transfer of plasma generated between two electrodes to the treated object through diffusion and convection mechanisms [4]. Since the direct and indirect NTP have been commonly used to treat living cells, tissues, DNA, and other biological targets, the experimental parameters of NTP (including gas composition and flow rate, frequency, voltage, power, discharge distance, treatment time, etc.) are critical to control the physical characteristics of plasma and produce the desired effects [4,5,6]. Furthermore, it has been shown that the addition of the appropriate level of catalyst into the NTP reactor device generates better results than the NTP treatment alone [7].

**Figure 1:**
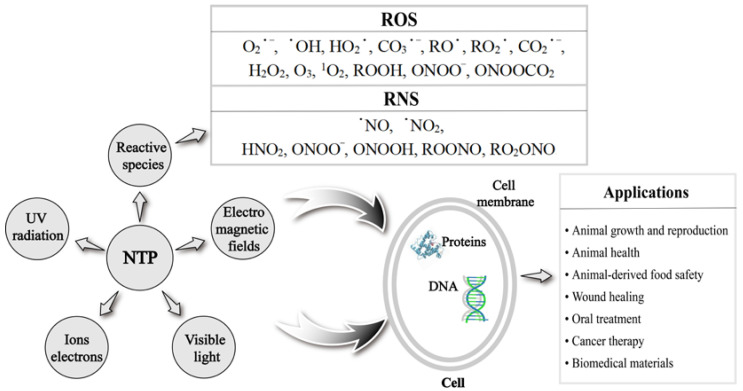
The main compositions of non-thermal plasma (NTP) and its applications.

NTP-generated reactive oxygen species (ROS), reactive nitrogen species (RNS), UV radiation, and high-energy electrons directly play an important role in many fields [7] (Figure 1). ROS mainly include oxygen-containing radicals and peroxides, such as superoxide (O_2_^•−^), hydroxyl (^•^OH), hydroperoxyl (HO_2_^•^), carbonate (CO_3_^•−^), alkoxyl (RO^•^), peroxyl (RO_2_^•^), carbon dioxide radical (CO_2_^•−^), hydrogen peroxide (H_2_O_2_), ozone (O_3_), singlet oxygen (^1^O_2_), organic peroxides (ROOH), peroxynitrite (ONOO^−^), and nitrosoperoxycarbonate (ONOOCO_2_). RNS is centered on nitric oxide (NO) and includes nitric oxide (^•^NO), nitrogen dioxide (^•^NO_2_), nitrous acid (HNO_2_), peroxynitrite (ONOO^−^), peroxynitrous acid (ONOOH), alkyl peroxynitrites (ROONO), and alkyl peroxynitrates (RO_2_ONO) [8]. NTP-produced radicals or reactive species can be short-lived or long-lived, and their lifetime is closely related to the effect and distance of their action. Short-lived radicals or species include O_2_^•−^, ^•^OH, O_3_, and ONOO^−^, etc. Long-lived radicals or species include H_2_O_2_, ^•^NO, and ^•^NO_2_, etc. [9]. All radicals or species in ROS except H_2_O_2_ are short-lived, so it is crucial to conduct in-depth studies on ROS and strictly control their levels. Before the NTP-produced radicals or species reach the cell interior, ROS need to traverse at least three major barriers at the plasma–fluid, tissue fluid–tissue, and tissue–cell interfaces [10]. During their passage through the physiological environment, ROS need to break the obstacles such as extracellular matrix proteins and antioxidants. However, how far NTP-generated ROS are delivered into real biological targets and how these ROS interact with the various components of a real tissue or cells (such as tissue fluid, extracellular matrix, cell membrane, and intracellular components, etc.) need to be clearly elaborated. In addition, ROS, which are cellular respiratory by-products produced by aerobic metabolism, play an important regulatory role in various physiological and pathological processes of animals as important intracellular messengers. Studies showed that NTP could stimulate intracellular ROS generation through interacting with cellular components [11,12,13,14,15] and regulate intracellular ROS levels indirectly through modulating redox balance-related transcription factors and signaling pathways [16,17,18,19,20]. ROS have the characteristics of a “double-edged sword”, and it is very important to analyze and control the levels of intracellular ROS required to achieve positive effects [21]. Low levels of intracellular ROS are essential for various biological functions, including cell survival, cell growth, cell proliferation and differentiation, and immune response, while excessive intracellular ROS cause damage to proteins, DNA, and RNA, which lead to genetic alterations in cells and induce cell apoptosis [22]. Therefore, clarifying the mechanisms of NTP-produced exogenous ROS directly, NTP-stimulated intracellular ROS, and NTP-regulated intracellular ROS indirectly are crucial for its applications.

NTP technology has the advantages of safety, non-toxicity, and high efficiency, which make it widely used in disinfection and sterilization [23], would healing [24], oral treatment [25], cancer therapy [26], and biomedical materials [27]. The last two decades have witnessed the rapid development of NTP technology, from theoretical and experimental research to practical applications in various fields. Evidence from recent studies indicate that most of the activity of NTP comes from the production of reactive species (Figure 1). It remains not well known how NTP generates exogenous ROS and stimulates or regulates intracellular ROS, which induce different effects on biological targets. In addition to the wide applications in biomedicine, NTP also shows a great application potential in animal husbandry. Direct NTP treatment was found to improve the growth rate and male reproductive capacity of poultry [16,17]. NTP can also improve the appearance and flavor of animal-derived food and even affect the composition of nutrients through sterilizing and changing the structure of food [28]. In addition, NTP shows great advantages in biological oxidation. Ahmadi et al. [29] found that NTP can oxidize D-glucose through inducing the production of active substances; this provides a direction for the development of new environmental technology in the future. NTP-generated ROS can induce the oxidation of sulfhydryl-containing amino acids (such as cysteine and methionine) [30,31]. Wang et al. [32] found that NTP-generated ROS accelerated the lipid oxidation of meat. Moreover, ROS can promote the secondary oxidation of lipids to produce aldehydes or shorter chain fatty acyl compounds [33]. The above effects were mediated by the experimental parameters of NTP [32], which brought challenges to the application of NTP in animal health. Evidently, applications of NTP-mediated ROS levels are becoming more and more promising. How to precisely control the ROS levels through modulating parameters of NTP treatment on different biological targets is the key issue to be solved in clinical practice.

Most of the current studies systematically illustrate the regulatory effects of NTP on reactive species and the applications of NTP, but the effects and mechanisms of NTP-mediated ROS are not well developed. Based on the impacts of ROS on animal health, this review attempts to illustrate the mechanism of NTP on mediating ROS levels from three aspects (direct generation of exogenous ROS, stimulatory generation of intracellular ROS, indirect regulation of intracellular ROS), and summarize the potential applications of NTP-mediated ROS in animal husbandry and biomedicine, which will provide a theoretical basis for the optimization of NTP technology and the development of advanced NTP devices.

## 2. Effects of ROS on Animal Health

Under physiological conditions, cells harmonize the production and removal of ROS via antioxidant systems. The physiological levels of ROS are different for different cells and tissues; the appropriate levels of ROS can maintain or promote cell function and homeostasis, while excessive ROS show less effective and cause oxidative stress damage which further induce cell death [34]. Oxidative stress damage usually occurs when cells and tissues suffer from the stimulation of environmental stress or adverse factors. The imbalance of oxidants and antioxidants can lead to a sharp increase in the level of ROS, which result in the destruction of biofilm, proteins, and DNA, and further damage the normal function of cells and tissues [35]. Therefore, ROS homeostasis is of great significance in regulating normal physiological processes of animals.

### 2.1. Mechanism of ROS Generation and Removal

Intracellular ROS are mainly derived from the cytoplasm and mitochondria. Mitochondria are not only the main site of energy production, but also the site of ROS production. Flavin mononucleotide (FMN) and ubiquinone in complex I, flavin adenine dinucleotide (FAD) in complex II, and Q_o_ site in complex III are currently considered to be the main site of ROS [36]. Electrons are transferred from sites of low redox potential to sites of high potential in the process of biological oxidation. Since complex I is low potential, the FMN site at the entrance of electron transport chains is highly susceptible to oxidation to produce ROS. Normally, complex II has no significant effect on ROS production, but in case of inhibition of complex I and III and low succinate concentration, complex II produces superoxide or H_2_O_2_ at a high rate [37]. The formation of ROS in complex III under normal conditions is mainly dependent on its characteristic electron transfer mechanism (the Q-cycle), whereas the excessive production of ROS by complex III may be due to acquired and genetic defects in the mitochondrial respiratory chain [38]. The cytoplasmic pathway of ROS production is achieved by the electron transfer of nicotinamide adenine dinucleotide phosphate oxidase (NOX). The NOX family is a protease that transfers electrons to oxygen through biological membranes to generate superoxide. When the nicotinamide adenine dinucleotide phosphate (NADPH) binds to the oxidase, electrons are sequentially transferred from the NADPH substrate to the FAD prosthetic group, and then transferred to the iron porphyrin ring in the transmembrane domain. As the final electron acceptor, oxygen is reduced into superoxide or H_2_O_2_ on the other side of the membrane [39].

The generation and removal of ROS are in a state of dynamic equilibrium in healthy animals. The removal of excessive ROS relies mainly on the regulation of antioxidant enzyme activities which are mediated by Kelch-like epichlorohydrin-associated protein 1 (KEAP1)-nuclear factor-E2-related factor 2 (NRF2)-antioxidant response element (ARE) [40]. Among the antioxidant enzymes, superoxide dismutase (SOD) can catalyze the dismutation of superoxide into O_2_ and H_2_O_2_, and catalase (CAT) can further decompose H_2_O_2_ into O_2_ and H_2_O. Increased activities of intracellular SOD and CAT were found to inhibit ROS levels and reduce cell apoptosis [41]. In addition, glutathione peroxidase (GPX) and peroxidase (PRX) can inhibit the progression of liver fibrosis through removal of H_2_O_2_ and reducing ROS levels [42].

### 2.2. ROS Homeostasis and Animal Health

ROS are mainly involved in multiple signaling pathways through redox modification to perform specific functions. ROS homeostasis is essential for the regulation of animal health. Low levels of ROS promote cell proliferation through the activation of mitogen-activated protein kinases (MAPKs), nuclear factor-kappa B (NF-κB), and phosphoinositide 3-kinase (PI3K)-protein kinase B (AKT)-mammalian target of rapamycin (mTOR) signaling pathways [34,40]. MAPKs include extracellular-regulated protein kinase (ERK), c-Jun N-terminal kinase (JNK), p38 kinase, and big mitogen-activated protein kinase 1 (BMK1). ERK is involved in cell proliferation and differentiation through stimulating growth factors. ROS can phosphorylate the epithelial growth factor receptor, which activates ERK through Raf/mitogen-activated proteins. Activated ERK is transferred from cytoplasm to nucleus, resulting in the activation of transcription factor-activating proteins and NF-κB, which further regulate the growth of cells [40]. ROS can promote the degradation of inhibitory factor α of NF-κB (IκBα) through the phosphorylation and ubiquitination of IκBα, which dissociate the NF-κB/IκBα complex in the cytoplasm and increase the level of active NF-κB in the nucleus, thus participating in the regulation of genes which are related to cell adhesion, differentiation, proliferation, and immunity [43]. Moreover, ROS can also directly activate PI3K and initiate the PI3K-AKT-mTOR signaling pathway, which is involved in cell proliferation, muscle development, and other processes [40]. Direct administration of low concentrations of exogenous ROS can promote the proliferation of endothelial cells and fibroblasts, and further accelerate wound healing, whereas interfering with intracellular ROS production inhibits wound closure [44]. In addition, ROS play a significant role in regulating the progression of cell differentiation. Hamanaka et al. [45] observed that the inhibition of ROS production using antioxidants resulted in inhibition of keratinocyte differentiation in vitro, while supplementation with exogenous H_2_O_2_ could partially alleviate this situation. ROS can also affect the level of cellular glycolysis through regulating the expression of hypoxia-inducible factors [46], and participate in various metabolic processes such as glycolysis, lipid metabolism, and autophagy through regulating the activity of adenosine monophosphate-activated protein kinase [47].

### 2.3. Excessive ROS and Animal Disease

High concentrations of ROS are essential for body defense and immune response under physiological conditions. When the body is invaded by pathogens, phagocytes generate high levels of ROS through “respiratory burst”, which kill pathogenic microorganisms via base modification or generation of ^•^OH and hypochlorite, thus achieving anti-viral, anti-bacterial, and anti-parasitic effects [48]. As a major second messenger within T-cells, ROS can activate T-cells and regulate cell proliferation via the MAPK pathway. High levels of ROS can promote the differentiation of Th2 cells and increase the production of IL-4 and IL-2 in T-cells [49]. Moreover, most T-cells are dependent on apoptosis, which is induced by high concentrations of ROS to maintain the internal environment homeostasis, with the exception of a small proportion of T-cells that turn into memory cells and survive [50].

High concentrations of ROS can increase the expression of pro-apoptotic proteins and open the mitochondrial permeability transport pores, resulting in a decrease in mitochondrial membrane potential. Cytochrome c combines with apoptotic protease activators to form apoptotic bodies, which induces cell apoptosis through activating the caspase pathway [40]. Excessive ROS have been found to contribute to the occurrence of diabetes through damaging pancreatic β-cells and reducing insulin sensitivity in peripheral tissues [51]. Hyperglycemia-induced excessive ROS promotes the ubiquitination degradation of the rate-limiting enzyme of tetrahydrobiopterin synthesis, which further leads to the inactivation of endothelial nitric oxide synthase and the damage of endothelium-dependent relaxation [52]. Excessive ROS can also inhibit the expression of vascular endothelial growth factor (VEGF) and the formation of neovascularization after ischemia, thus decreasing the differentiation capacity of bone marrow-derived monocytes and endothelial progenitor cells [53]. ROS-mediated signaling pathways were found to play an important role in promoting the dysregulation of vascular tone and the formation of impaired neovascularization [54]. ROS levels in cancer cells are much higher than those in normal cells because of redox imbalance and increased levels of oxidative stress, which makes cancer cells more susceptible to cell death when subjected to oxidative stress [26]. This suggests that strengthening the body’s antioxidant capacity or avoiding the generation of oxidative stress can prevent and treat cancer-related diseases to a certain degree.

## 3. Mechanisms of NTP-Mediated ROS

### 3.1. Direct Generation of Exogenous ROS by NTP 

**Figure 2:**
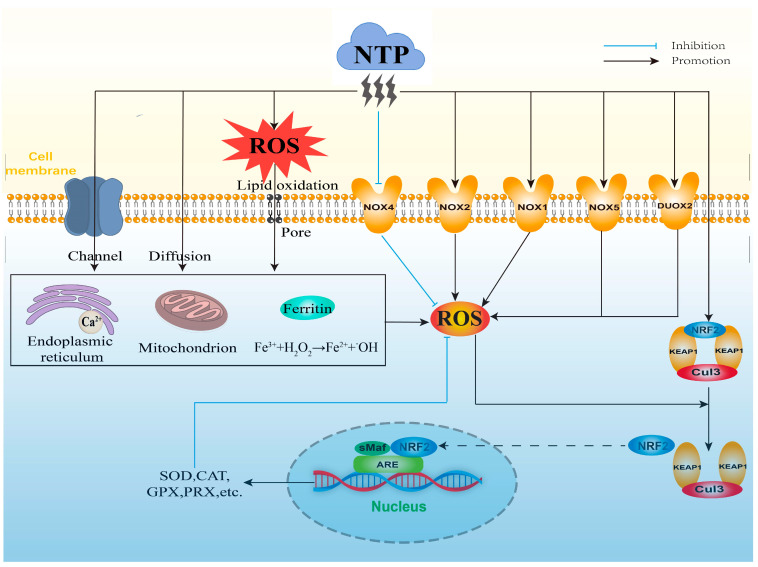
The potential mechanisms of NTP on mediating ROS levels.

ROS are usually generated at the boundary of the plasma jet with the adjacent air [55]. When the NTP discharge is excited, ROS are generated by various collisions and energy exchange processes between electrons, atoms, and neutral gas molecules (Figure 2) [56]. Since NTP can produce exogenous ROS in a time- and intensity-dependent manner, the concentration of ROS is related to the experimental parameters of NTP. Vandamme et al. [57] found that the level of H_2_O_2_ generated by direct treatment with 20 J/cm^2^ of NTP was higher than that generated by treatment with 10 J/cm^2^ of NTP, and Adhikari et al. [58] found that ROS generated by air-mixed argon plasma injection increased the levels of ROS. Although most of the current studies have focused on the application of NTP to regulate intracellular ROS levels, there are still some studies showing that exogenous ROS generated by NTP can also play a role to a certain extent. For example, Han et al. [59] found that exogenous ROS could lead to cellular rupture and slight DNA damage, which consequently inactivated *E. coli*. Further, the exact radical or reactive components produced by different types and parameters of NTP and their concentrations and lifetimes need to be clarified for better understanding the direct effects of NTP on biological targets. 

### 3.2. Stimulatory Generation of Intracellular ROS by NTP

The mitochondrial respiratory chain is one of the most important sources of ROS generation in cells. Zhang et al. [11] found that appropriate NTP treatment could stimulate the moderate production of intracellular ROS through increasing the number of mitochondria and the activity of mitochondrial respiratory complexes (Figure 2). NTP treatment may also slow down the electron flow through decreasing membrane potential, membranous interruptions, and alteration of a variety of mitochondrial matrix dehydrogenases, which promote the leakage of electrons and the increase of superoxide production [12]. In addition, activation of the mitochondrial respiratory chain is closely related to calcium (Ca^2+^) homeostasis, and Zhunussova et al. [12] found that NTP treatment increased intracellular Ca^2+^ concentration, which may facilitate the generation of intracellular ROS [21]. Moreover, NTP treatment can stimulate intracellular ROS production by affecting ferritin, which has a role in sequestering Fe^2+^ under physiological conditions and can block the binding of Fe^2+^ with H_2_O_2_ to produce ^•^OH. NTP-treated cancer cells revealed that some of Fe^3+^ that were stored in ferritin were reduced to free-catalyzed Fe^2+^, which further initiated the Fenton reaction and induced the generation of ^•^OH (Figure 2) [13]. In addition, exogenous ROS generated by NTP can induce the generation of intracellular ROS after entering into the cells [14]; this fact was supported by the study findings of Bekeschus et al. [60]. They compared the degree of cellular damage in NTP treatment with and without the addition of H_2_O_2_ enzyme, and found that the addition of H_2_O_2_ enzyme prior to the NTP treatment caused the effect of NTP to be completely eliminated, and that the addition of H_2_O_2_ enzyme after the NTP treatment caused the effect of NTP to be weakened [60]. Appropriate conditions of NTP treatment produce low levels of exogenous ROS that can enter cells or tissues through the method of passive diffusion, membrane channels, or pores created by lipid oxidation on cell membranes (Figure 2) [10]. Moreover, Ahn et al. [15] also found that the extracellular ROS generated by NTP could diffuse into the cells and stimulate the generation of intracellular ROS, which is of great significance for the design of anti-cancer drugs and the research of therapeutic strategies. However, the stimulatory effect of intracellular ROS by NTP-produced exogenous ROS should consider their lifetime and physical barriers during their delivery into cells [10]. Therefore, how far NTP-produced exogenous ROS are delivered into cells and how these ROS generate intracellular ROS, and how NTP interacts with the various cellular components to stimulate generation of intracellular ROS need to be further investigated for better distinguishing the biological effect of NTP through direct exogenous ROS or stimulatory generation of intracellular ROS. 

### 3.3. Indirect Regulation of Intracellular ROS by NTP

In addition to the above mechanisms, NTP can also indirectly regulate intracellular ROS levels through modulating redox balance-related transcription factors and signaling pathways (Figure 2). The NOX family is a protease that generates superoxide during the process of electron transfer to oxygen through biofilms, and it consists of seven isoforms (NOX1, NOX2, NOX3, NOX4, NOX5, DUOX1, and DUOX2). NTP treatment is capable of regulating intracellular ROS levels in an intensity- and time-dependent manner. Appropriate conditions of NTP treatment produce low levels of exogenous ROS which are transferred into the cells that can affect intracellular ROS production through inhibiting mRNA expression of NOX4. This attenuates the formation of peroxides during the process of electrons transfer from NADPH to oxygen molecules [61,62]. NTP treatment can also increase intracellular ROS generation through up-regulating the mRNA expression of NOX2. Ishaq et al. [19] observed that NTP exposure significantly reduced intracellular ROS generation in the NOX2-silenced Caco-2 human colorectal cancer cells. In addition, NTP treatment induces the expression of the NOX family and regulates the generation of intracellular ROS via epigenetic modification. Kang et al. [20] observed that DBD-treated human keratinocyte HaCaT cells showed higher expressions of NOX1, NOX5, and DUOX2, and higher levels of intracellular ROS, which resulted from the regulation of DNA methylation and histone modification.

The KEAP1-NRF2-ARE antioxidant signaling pathway plays a key role in the removal of cellular ROS and protection from oxidative stress damage. NRF2 is the major regulator in the KEAP1-NRF2-ARE antioxidant signaling pathway. Under physiological conditions, NRF2 forms a dimer with KEAP1, which inhibits the activity of NRF2 via the degradation of ubiquitin-proteasome. This maintains NRF2 at a low level and to be anchored in the cytoplasm by KEAP1 [63,64]. ARE is a cis-acting enhancer found within the promoter region of many cytoprotective antioxidant genes [65]. Transcriptional activation of the ARE is primarily dependent on NRF2 stabilization, accumulation, and nuclear translocation through its dissociation from the cytoskeleton-associated KEAP1 [66]. When endogenous ROS are overproduced, the conformation of KEAP1 is altered by specific sulfhydryl modification, which results in the uncoupled reaction of NRF2 with KEAP1 and E3 ubiquitin ligase Cullin 3 (Cul3). The dissociated NRF2 is transferred into the nucleus and combines with the intra-nuclear sMaf protein to form a heterodimer, which binds to ARE on the promoter of target genes, and further initiates the expression of down-stream antioxidant enzymes (such as GPX, SOD, CAT, etc.) [67], thus maintaining intracellular ROS at an appropriate level (Figure 2). It has been found that ROS regulated by appropriate NTP exposure conditions can activate the KEAP1-NRF2-ARE signaling pathway in a time- and intensity-dependent manner, which further regulates the intracellular ROS homeostasis through increasing the activities of antioxidant enzymes in the testis of roosters, subsequently affecting Sertoli cell proliferation and sperm quality [16,17]. NTP can also affect the levels of intracellular ROS via the direct activation of NRF2. Schmidt et al. [18] showed that silence of NRF2 expression using small interfering RNA (siRNA) can re-activate NRF2 and up-regulate the activity of ARE-dependent target genes after the treatment of NTP. In addition, DNA methylation can affect the intracellular ROS levels through regulating the expression of antioxidant genes. Appropriate NTP treatments reduce the transcription levels of KEAP1 and activate the activities of NRF2 and antioxidant enzymes through increasing the CpG methylation of KEAP1 at its promoter region, which decreases the levels of intracellular ROS [68]. However, high-intensity NTP treatments lead to excessive levels of intracellular ROS through inducing hypermethylations of KEAP1 and antioxidant enzyme genes at their promoter regions [62]. Therefore, NTP treatment under appropriate conditions is essential for its positive effects through regulating the generation and removal of cellular ROS. 

In brief, NTP can mediate ROS levels via the above three aspects. These are NTP-produced exogenous ROS directly, NTP-stimulated intracellular ROS generation mainly through interacting with cellular components, and NTP-regulated intracellular ROS indirectly through modulating redox balance-related transcription factors and signaling pathways; however, there are still many gaps to be filled. A comprehensive understanding of the mechanisms by which NTP mediates ROS will help to better exploit the positive effects of NTP technology.

## 4. Applications of NTP-Mediated ROS

### 4.1. Application of NTP in Animal Husbandry

Animal husbandry is an important industry related to the economy and people’s livelihood. In order to accelerate its development, current research mainly focus on improving the breeding capacity of livestock and poultry and guaranteeing the safety of animal-derived food. The development of animal husbandry is limited because of the increasing environmental pollution and the impact of epidemics in recent years. Therefore, exploring new strategies and technologies to promote the stable advancement of animal husbandry is a powerful initiative for solving those problems.

#### 4.1.1. NTP Improves the Growth and Breeding Ability of Animals

The most fundamental measure to promote the healthy development of animal husbandry is to improve the growth and reproductive capacity of livestock and poultry. It was found that cell proliferation and limb development were promoted in low doses of NTP-treated chicken embryos at different embryonic stages [11]. On the one hand, appropriate NTP treatment can ensure the normal physiological activities of livestock and poultry through affecting ATP levels and maintaining ROS homeostasis; on the other hand, NTP treatment can regulate the demethylation level of growth-related hormone synthesis genes and energy metabolism-related genes in skeletal muscle and the thyroid gland, and increase the expression of growth hormone and related factors, resulting in the increase of chicken growth rate [11,17]. However, high doses of NTP treatment can affect the antioxidant signaling pathway in a dose-dependent manner, which results in excessive accumulation of ROS and cytotoxicity, leading to the death of chicken embryos [69].

Sperm quality directly affects the reproductive performance of livestock and poultry, and determines the hatching rate of fertilized eggs and the survival rate of offspring. ROS levels are within the physiological dose range because of the antioxidant enzyme system in testis, which stimulate sperm capacitation and acrosome reaction to ensure successful fertilization. However, a decreased antioxidant capacity can lead to an imbalance of ROS levels, which induces oxidative stress damage and further reduces sperm quality and fertilization ability [70]. Zhang et al. [16,17] observed that appropriate NTP treatment regulated ROS homeostasis through increasing the activities of antioxidant enzymes in rooster testis. Meanwhile, it improved sperm motility through increasing ATP levels and activities of mitochondrial respiratory enzymes. However, high-dose NTP treatment caused a large amount of ROS accumulation in spermatozoa. On the one hand, excessive ROS impaired the NRF2 antioxidant signaling pathway, leading to lipid peroxidation of sperm membranes; on the other hand, it caused sperm mitochondrial dysfunction, blocking ATP production and damaging sperm axons. The combined action of ROS-induced effects impaired sperm motility and acrosome integrity, which further inhibited sperm capacitation and acrosome reaction [62]. NTP has potential applications in improving the growth and reproductive performance of poultry through regulating the ROS levels. In-depth research on optimizing the conditions of NTP treatment and expanding the scope of its application will provide a direction for the future development of animal husbandry.

#### 4.1.2. NTP Improves the Animal Health

Reducing the contamination of the livestock and poultry feeding environment plays a key role in improving animal health. Microorganisms in the environment increase the risk of disease in livestock and poultry through infection and transmission. NTP technology has been widely used in biological decontamination and improvement of the feeding environment. It can effectively kill microorganisms in the environment, such as *Escherichia coli* (*E. coli*), *Salmonella*, and even *Staphylococcus aureus* (*S. aureus*) and *Pseudomonas aeruginosa* which are highly resistant to antibiotics (Table 1). The bactericidal mechanism is related to the levels of ROS induced by NTP [71]. The bactericidal ability of NTP on microorganisms is mainly related to NTP-generated reactive species and the changes of ROS in microorganisms. Yahaya et al. [72] showed that direct NTP treatment using air as a working gas showed a better sterilizing effect and higher ROS level. The oxidative damage effects of NTP-generated ROS on Gram-negative and -positive bacteria are different because of their different bacterial structures. Gram-positive and Gram-negative bacteria which were treated with DBD (230 V, 50 Hz) for 5 min showed different effects; Gram-negative bacteria had severe perforation on the cell membrane, while Gram-positive bacteria had only partial shrinkage or slight deformation [73]. The mechanism is mainly that lipopolysaccharides in the outer membrane of Gram-negative bacteria are highly sensitive to ROS, and the unsaturated C=C in their phospholipid components can be peroxidized by ROS, which further impair the structure of the bacterial membrane and achieve the bactericidal effect. However, Gram-positive bacteria are less sensitive to ROS because of the protective effect of their peptidoglycan structure [74]. Although it has been proven that NTP treatment has a good inactivation effect on bacteria in a planktonic state, most microorganisms exist in the environment in a biofilm state. The formation of biofilms makes it extremely difficult to inactivate microorganisms. Compared with the conventional sterilization methods which are not very efficient, DBD treatment shows excellent effects on the inactivation of *E. coli* and *S. aureus* biofilms [75].

NTP treatment can remove harmful pollutants from air and water through regulating ROS levels. For harmful gases, ROS generated by NTP can collide with gas molecules to excite them to a higher energy level [76]. The internal energy of the excited gas molecules increases continuously, resulting in the breaking of chemical bonds and chemical reactions with other substances. Eventually, organic molecules are oxidized and degraded into small molecule compounds with harmless or less toxicity, which allow pollutants to be decomposed or transformed in a short period of time, resulting in the removal of harmful substances from the air [77]. In addition to excrement and feed residues, wastewater from livestock and poultry farms contains various pharmaceuticals which are harmful to animal health. Although traditional treatment methods have purified the wastewater to a large extent, most of the pollutants still cannot be degraded. NTP-generated ROS can cause bond breaking and ring opening of unsaturated bonds in drugs, which is followed by gradual decomposition into small molecules. This helps to improve the overall biochemistry of refractory substances and thus significantly promotes the purification of wastewater [78]. In addition, NTP treatment has a degradation effect on antibiotics in wastewater. Li et al. [79] found that NTP-generated ROS can effectively degrade penicillin and reduce antibiotic pollution in the environment. Consequently, NTP-mediated ROS levels have advantages in purifying the environment, ensuring water purification, and degrading antibiotics, which maximize the improvement of livestock and poultry health and enhance the economic benefits of animal husbandry.

#### 4.1.3. NTP Ensures the Safety of Animal-Derived Foods

Animal-derived foods are highly susceptible to microbial contamination during processing, transport, and packaging, resulting in many safety problems such as spoilage, that seriously endanger human lives and health. Traditional methods of meat preservation have limitations to a certain extent, such as thermal sterilization technology that alters the structure and texture of meat [80]. Recent studies have shown that the highly active substances produced by NTP can inactivate microorganisms and thus play a role in preservation without damaging food nutrition and quality. NTP-produced ROS can destroy the morphology and integrity of microorganisms on meat products [81]. In addition to damaging bacterial DNA, proteins, and enzymes, ROS may induce the formation of unsaturated fatty acids peroxides and the oxidation of amino acids in proteins through interacting with lipids [80]. The capacity of NTP treatment to ensure the safety of animal-derived foods is closely related to its types. The closer distance between direct NTP and object results in the greater inactivation of microorganisms, but the higher intensity of exposure may cause adverse effects on the surface of meats (e.g., burn marks) [82]. Therefore, the types and treatment parameters of NTP are critical for meat preservation. NTP-mediated ROS levels have also shown great advantages in the preservation of dairy products. Thermal pasteurization is usually used to kill microorganisms for ensuring the safety of milk, but the temperature and time must be strictly controlled, otherwise it will affect the physicochemical properties and nutritional quality of milk. It was found that NTP treatment (DBD-CP, voltage from 70 to 80 V, treatment time of 120 s) on milk could destroy the DNA of bacteria such as *S. aureus* and *E. coli* to achieve the sterilization effect without changing the color, pH, and quality of milk. This indicates that NTP technology can be used as an innovative method for milk sterilization [83]. Korachi et al. [84] observed that the sterilizing effect of *E. coli* in milk using the NTP corona discharge system was relatively considerable (Table 1). Although several volatile compounds were produced, no significant changes in the lipid composition of milk were observed. In addition, NTP is effective in the sterilization and preservation of eggs. The number of *Salmonella* on eggs was found to be significantly reduced in a plasma-activated water (PAW) treatment group (Table 1), and the mechanism was related to PAW-produced ROS levels [85]. 

Moreover, the advantages of NTP on sterilization can be used in the packaging of animal-derived foods. NTP treatment on packaging bags which are used for fresh pork showed a significantly reduced number of *E. coli* and *Salmonella typhimurium* in pork [86] (Table 1). The gas molecules inside the packaging bag collide with the high-energy electrons produced by NTP to generate ROS, which inactivate the microorganisms on the packaging bag and animal-derived foods, thus effectively extending the shelf life of food [80]. Therefore, NTP treatment plays a sterilizing effect via the effective regulation of ROS levels, which help to solve the problem of freshness through its effects on food itself or packaging, to extend the shelf life of animal-derived foods and protect food safety. 

Although it provides a new direction for NTP technology application in agriculture and food production, there are still many challenges to the widespread application of NTP [87,88]. On the one hand, NTP technology applied in agriculture and food production is currently limited to the laboratory level. If it is extended to the large-scale commercial market, the cost-effectiveness will be greatly affected, and the demand for human resources will be increased. On the other hand, NTP treatment needs to be optimized with strict experimental parameters in order to achieve the desired results, because the different NTP devices and treatment conditions can significantly affect the quality and safety of food products. In addition, there is currently no evidence to show potential toxicity of NTP to human beings and its side-effects on food. As for the regulatory aspect, there is currently no corresponding policy and technology in the market to monitor the residues of NTP-generated reactive species in food. If NTP technology is widely extended to the market, it may give rise to the misuse of NTP which could induce massive residues in food and even cause side-effects, and even illegal activities (e.g., reprocessing of food products that have exceeded their shelf-life and re-placing them on the market), this will impose a heavy burden on the work of food regulatory authorities. Therefore, it is essential to accelerate the improvement of NTP treatment condition optimization, and to assess the risks of NTP technology in food and human beings. Moreover, it is very important to improve policies and techniques for monitoring NTP residues, and to develop regulations on food production and processing, in order to put NTP technology into the agricultural and food markets.

**Table 1:** Application of NTP on sterilization through mediating ROS levels at different treatment parameters.

Bacterium	Treated Sample	Plasma Generating Source	Treatment Parameters				Inhibition Rate
			Frequency	Voltage	Time	Gas	
*Staphylococcus aureus*	Suspension on Petri dish [72]	DBD	27 kHz	900 V	3 min	air	99%
	Biofilm in plates containing TSB medium [89]	DBD	22 kHz	7 kV	4 min	air	70%
	Sample solution in Petri dish (combination with ultrasound) [90]	DBD	10 kHz	14 kV	NTP 5 minUS 20 min	air	100%
*Pseudomonas aeruginosa*	Suspension on Luria–Bertani agar plates [91]	plasma jet	*	10 kV	10 min	air	more than 88%
	Suspension on Mueller–Hinton agar [92]	plasma jet	20 kHz	6 kV	2 min	helium: oxygen (99.5%: 0.5%)	more than 75%
	Suspension on Luria–Bertani agar plate [93]	plasma jet	20 kHz	14.7 kV	60 s	air	more than 40%
*Escherichia coli*	Suspension in a glass container [94]	DBD	25 kHz	8.5 kV	15 s	air	28%
	Suspension in filtered PBS [95]	DBD microfluidic plasma reactor	17 kHz	10 kV	5.3 s	air	100%
	Exists in vacuum packaging of pork butt [86]	DBD	30 kHz	3 kV	10 min	nitrogen: oxygen (99.9%: 0.1%)	43%
	Suspension into sterile milk in a sterile Petri dish [84]	corona discharge	50 kHz	9 kV	3 min	air	54%
*Salmonella*	Suspension in sterile saline solution [96]	DBD	2.5 kHz	3 kV	5 min	air	more than 70%
	Inoculated on shell eggs [85]	plasma jet	16 kHz	3 kV	90 s	air	more than 60%
	Exists in vacuum packaging of pork butt [86]	DBD	30 kHz	3 kV	10 min	nitrogen: oxygen (99.9%: 0.1%)	49%

*, unspecified parameter.

### 4.2. Application of NTP in Biomedicine

Plasma medicine is a rapidly developing interdisciplinary field of research that combines plasma physics and life sciences, enabling plasma to be applied directly to or in the human body for therapy [97,98]. The first NTP devices were certified as CEIIa grade medical devices for the treatment of chronic wounds and pathogenic skin diseases in Germany and Europe in 2013. Compared with conventional treatments, NTP treatments have no significant negative effects on healthy tissues, and the manufacturing costs of NTP-generating devices are relatively low [5]. Therefore, the high potential of NTP for medical applications has received increased attention in recent years, and it has been widely used in wound healing, oral treatment, cancer therapy, biomedical materials, and other fields [99,100,101]. Notably, the appropriate dose of NTP treatment must be strictly controlled under the guidance of experts in order to avoid potentially harmful effects in clinical practice.

#### 4.2.1. NTP Promotes Wound Healing

**Figure 3:**
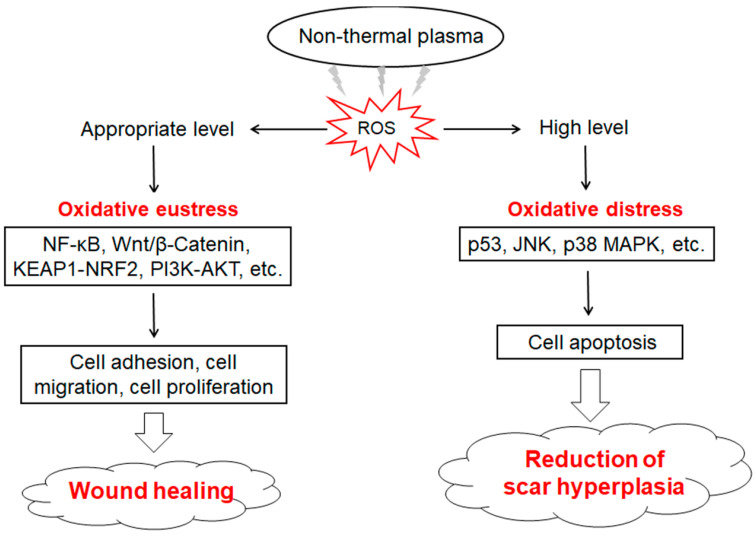
Effects of NTP-generated ROS on wound healing and scar treatment. Low/appropriate NTP treatments produce a physiological level of ROS, which activates Wnt/β-Catenin, Kelch-like epichlorohydrin-associated protein 1 (KEAP1)-nuclear factor-E2-related factor 2 (NRF2), phosphoinositide 3-kinase (PI3K)/protein kinase B (AKT), and nuclear factor kappa-B (NF-κB) signaling pathways that promote cell adhesion, migration, and proliferation, and further improve wound healing. High NTP treatments produce a high level of ROS, which induces cell apoptosis through regulating p53, c-Jun N-terminal kinase (JNK), and p38 mitogen-activated protein kinase (MAPK)-mediated caspase pathways, and further suppress hypertrophic scar formation.

There are four main stages of wound healing: hemostasis, inflammation, proliferation, and remodeling. Traditional hemostatic methods such as electrocautery are prone to causing adverse reactions in patients, and internal bleeding can happen easily several hours after surgery. Bekeschus et al. [102] developed a medical gas plasma jet technology for blood coagulation and demonstrated that it interferes with the biochemical reactions during coagulation. NTP-produced reactive species (e.g., ROS and RNS) promote platelet activation and fibrin filament formation through changing the activity of relevant molecules [103]. Striesow et al. [104] investigated the mechanism of ROS-promoted coagulation through treating washed platelets with plasma which contained ROS components. Bekeschus et al. [105] confirmed that ROS could activate platelets through improving the expression level of P-selectin on the outer membrane of platelets, and the ROS-activated effects on platelets showed significant differences in different treatment time and plasma composition. Keratinocytes, fibroblasts, vascular endothelial cells, and inflammatory cells are closely associated with the process of wound healing. Evidence has shown that NTP treatment can accelerate wound healing through regulating the biological effects of ROS, which promote skin cell proliferation and angiogenesis under a lower intensity of NTP [101,106]. Cellular redox imbalance is an important reason for the difficulty of wound healing. ROS induced by appropriate conditions of NTP exposure can enhance the antioxidant capacity of cells and reduce tissue damage and inflammation through activating the KEAP1-NRF2-ARE signaling pathway [18,107]. Activated KEAP1 promotes actin cytoskeleton structural reorganization and local adhesion, and increases granulation tissue formation and matrix deposition, while NRF2 significantly up-regulates VEGF expression and induces endothelial cell proliferation and migration, which help to promote neovascularization [108]. As a key downstream molecule of p53-regualted cell cycle and glutathione metabolism, NRF2 enhances the cellular antioxidant capacity [109]. Wu et al. [110] found that low-dose NTP-generated ROS accelerated wound healing through enhancing keratinocyte activity. This was induced by increased mitochondrial membrane potential or promoting keratinocyte migration and proliferation which were activated by the Wnt/β-Catenin and PI3K-AKT-mTOR signaling pathways. ROS induced by lower intensity and shorter duration of NTP treatment can activate the NF-κB signaling pathway which up-regulates the expression of G1/S-specific Cyclin D1 (Cyclin D1) and further promotes the proliferation of fibroblasts [111]. However, the higher intensity and longer duration of NTP treatment causes excessive accumulation of intracellular ROS, which lead to the apoptosis of fibroblasts [112]. Therefore, NTP has a potential application in inhibiting the over-proliferation of cutaneous scar tissues (Figure 3). 

Heinlin et al. [113] conducted a study of patients with donor skin grafts on the thigh and showed that patients in the NTP treatment group had a much better result of wound healing compared to the placebo group from the second post-treatment day. Landscheidt et al. [114] observed that the application of an active wound dressing that was treated with NTP could accelerate the process of wound re-epithelialization and reduce bacterial infection without any adverse effects, whereas repeated surgical wound debridement did not allow the wound to heal completely. Bacterial contamination is one of the major factors that impedes the healing of skin wounds. NTP exposure can reduce the ROS concentration in bacteria, thereby disrupting the function of bacteria [91]. In particular, it is noted that there are currently no recognized microorganisms which are resistant to NTP treatment. Mirpour et al. [115] found that NTP treatment sufficiently reduced bacterial load and promoted healing of diabetic foot ulcers without significant side effects on normal tissue. Therefore, NTP, as an emerging biomedical treatment strategy, is expected to be widely applied in wound repair and tissue regeneration [116]. However, the detailed mechanism of interactions between NTP and cells, NTP and tissues, and their side effects need to be further explored in the future.

#### 4.2.2. NTP Used in Oral Treatment

NTP has characteristics of safety, painlessness, and efficiency in oral treatment. Seo et al. [117] found that the bactericidal effect in the oral cavity lasted for a period of time after NTP treatment, and NTP-produced ROS were able to maintain the viability of gingival cells and make gingival fibroblasts to have a stronger antimicrobial activity in vivo. Bacterial infection is one of the main reasons for root canal treatment failure. NTP can enter the narrow dentin tubule to kill *Enterococcus faecalis*, which is a major pathogenic bacterium for surgical failure, and the bactericidal effect of NTP is significantly higher than that of traditional treatments such as calcium hydroxide and chlorhexidine [118]. In addition to being used for sterilization, NTP can be used to disrupt the drug-resistant dental biofilms safely and effectively [119]. Dental biofilms are a mixed microbial community which are firmly attached to the tooth surface and embedded in a polysaccharide and protein matrix [120]. This structure of dental biofilms makes bacteria which have biofilm capacity more resistant to host immune defense system, antibiotics, and antimicrobial agents. Therefore, removal of dental biofilms is paramount to the successful treatment of mucosal damage around implants in clinical practice. NTP-induced ROS can cause oxidative damage to mitochondrial DNA, proteins, and lipids, which disrupt the function and integrity of cells, and further lead to the inactivation of biofilms [75]. Jablonowski et al. [121] found that NTP-produced ROS effectively removed the naturally growing biofilm on the tooth surface, showing similar and even better bactericidal effects than the method of mechanical treatment. Therefore, NTP technology shows a great potential for application in oral treatment.

#### 4.2.3. NTP Contributes to Cancer Therapy

##### Application of NTP Alone in Cancer Therapy

Cancer has become one of the greatest threats to human life and health. Recently, an increasing number of studies have shown that NTP technology is a potential strategy for the treatment of cancers [122]. It has been shown that NTP can effectively kill cancer cells and induce apoptosis of lung cancer cells [123], pancreatic cancer cells [124], and thyroid cancer cells [125], etc. In vitro studies have shown that NTP-produced ROS were transferred into the cancer cells and further caused intracellular ROS accumulation, which were followed by DNA damage, decreased viability, and cell apoptosis [126,127,128,129]. Moreover, in vivo studies found that NTP treatment reduced the tumor volume and increased the weight and survival rate of mice [128,129,130] (Table 2). NTP-mediated ROS induced apoptosis of cancer cells through increased expression of pro-apoptotic genes and decreased expression of anti-apoptotic genes, and had a stronger inhibitory effect on cancer cells than on normal cells. This may result from different adhesive behaviors and sensitivities of cancer cells and normal cells [26]. Zhou et al. [123] found that NTP-produced ROS inhibited the ERK signaling pathway in a time-dependent manner, thereby inducing apoptosis of lung cancer cells. In addition, ^1^O_2_ produced by NTP can be effectively applied in cancer therapy by affecting the activity of phospholipase A_2_ [131]. Moreover, plasma-activated medium (PAM) shows a good anti-cancer effect. Zhen et al. [124] found that ROS induced by PAM could down-regulate the expression of BCL 2, depolarize the mitochondrial membrane, and then activate the caspase reaction to induce apoptosis of pancreatic cancer cells; PAM-regulated ROS could also promote apoptosis of cancer cells through inhibiting the AKT signaling pathway, signal transduction, and activation of transcriptional protein. Jung et al. [125] found that ROS produced by PAM up-regulated the expression of early growth factors and increased the expression of growth inhibition and DNA damage-induced genes, and further resulted in the apoptosis of thyroid cancer cells. More notably, it has recently been shown that ROS can not only directly kill cancer cells, but also induce anti-cancer immune responses, which may be effective in inhibiting the metastasis and recurrence of tumors [132,133].

##### Application of NTP Combination in Cancer Therapy

NTP treatment can not only induce apoptosis of cancer cells, but also improve the drug sensitivity of chemo-resistant cancer cells such as glioblastoma cells [134]. Combining NTP and chemotherapy enhanced the effect of cancer treatment [135]. Van Loenhout et al. [136] found that NTP treatment increased exogenous ROS, and NTP treatment combination with the use of Auranofin inhibited the endogenous antioxidant defense system. This led to intracellular ROS accumulation, cell death, and immunogenic responses in glioblastoma. Boeckmann et al. [137] also demonstrated that the combination of Sm837 with NTP exerted a significant synergistic effect in the treatment of skin cancer. In addition, the combination of NTP and radiotherapy can selectively induce cancer cell apoptosis and inhibit the growth of cancer cells in vivo via the production of excessive ROS. Kenari et al. [138] confirmed that the combination of NTP and radiotherapy was more effective in inhibiting the growth of cervical cancer cells than that of radiotherapy alone, but there was no significant difference when compared with the efficacy of NTP treatment alone. However, the treatment parameters of NTP and the specific mechanism of its action require further investigation [26].

Systemic toxicity is one of the most difficult issues to address in clinical treatment with anti-cancer drugs. Traditional anti-cancer drugs cannot directly target cancer cells, but tend to increase the toxicity of normal cells, which make their efficacy much lower than that of in vitro experiments. Therefore, in recent years, to reduce the adverse effects caused by anti-cancer drugs, a large number of studies have been focused on targeting drugs specifically to cancer cells or designing prodrugs that are selectively activated by the characteristic factors of cancer cells. Compared to normal cells, cancer cells have a significant difference in the surrounding microenvironment due to the characteristics of uncontrolled growth and abnormal gene expression [139]. Therefore, this difference provides a variety of ideas for the design of prodrugs, such as the overexpression of enzymes which can provide a basis for enzyme prodrug therapy [140]. It has been demonstrated that the levels of ROS play an important role in the activation of prodrugs. The structures of prodrugs usually have active functional groups such as -OH, -NH2, or -CONHOH, which are masked by protective groups and activated by cleavage at the target sites [141,142]. Arylboronic acid and its esters were found to be ROS-sensitive protective groups in prodrugs, their protective effect lose in the presence of ROS [143]. It has been shown that NTP can generate ROS in an intensity- and time-dependent manner and deposit ROS around cancer cells. It further selectively activates the prodrugs through changing the structure of protective groups. Ahmadi et al. [139] found that the novel boronate ester type (5-FC) prodrug could be activated by NTP-induced reactive species, in which ROS played a key role in activating the prodrug under the Ar/H_2_ discharge regime. They also found that NTP-generated ROS activated the boronate pinacol ester fenretinide prodrug via the oxidative cleavage of boron carbon bond [142]. The effect of ROS-activated prodrugs depends on the treatment parameters, thus, it is crucial to strictly control the conditions of NTP treatment.

In addition, it has been found that NTP-induced repetitive oxidative stress situations contribute to identifying potential targets for overcoming anti-cancer drug resistance [144]. However, the above studies still have some limitations, especially the short-life and weak penetration of ROS, which limit the widespread application of NTP in cancer therapy. To deal with these problems, Freund et al. [145] demonstrated that NTP treatment on Ringer’s lactate and sodium chloride facilitated the prolonged storage of ROS, which enable NTP to break through the limitations in the treatment of cancers. Gugin et al. [146] also confirmed that screening and strictly controlling the treatment parameters of NTP improved the inhibitory efficiency on cancer cells through increasing the ability of ROS to penetrate cell membranes. In order to improve the accuracy and efficiency of NTP therapy, some novel NTP-responsive devices have been recently developed. To avoid cancer recurrence after surgical resection, Chen et al. [147] demonstrated that treatment on residual cancer cells at the surgical cavities with a local portable air-fed cold atmospheric plasma device effectively induced cancer immunogenic cell death in situ and evoked strong T-cell-mediated immune responses against the residual cancer cells. However, whether the anti-cancer effect of NTP-responsive devices has a relationship with ROS need to be further investigated.

In brief, for the application of NTP technology on cancer therapy in the future, the treatment parameters of NTP should be optimized according to the selective and accurate inhibitory effect of different levels of ROS which are mediated by NTP on cancer cells and normal cells, and the extensive combination of NTP with other therapeutic methods is conducive to achieve better anti-cancer effects. Moreover, effective NTP-responsive devices need to be developed for different cancer types and reduction of cancer recurrence. 

**Table 2:** Effect of NTP on different cancers through mediating ROS levels.

Cancer Category	Method	Cell Lines or Models	Result
Lung cancer	In vitro	CALU-1, SPC-A1	Inhibit cancer cell proliferation and migration, induce cancer cell apoptosis and necrosis [123]
Glioblastoma	In vitro In vivo	U-87 MG, LN-229, T98G SB28 (subcutaneous injection into female C57BL/6J mice)	Inhibit the growth and proliferation of glioblastoma cells, induce cancer cell apoptosis, decrease tumor volume, increase the survival rates of GBM-bearing mice [136]
Pancreatic cancer	In vitro	Aspc1	Inhibit cancer cell metastasis and proliferation, induce cancer cell apoptosis and autophagy [124]
Thyroid cancer	In vitro In vivo	BCPAP, HTh7, KTC2, 8505C, FRO-Luc FRO-Luc (subcutaneous injection into BALB/c-nude mice)	Cause mitochondrial dysfunction in cancer cells, induce cancer cell death [125]
Human colon cancer	In vitro	HCT116	Increase DNA damage, cause cancer cell death [126]
Anaplastic squamous cell carcinoma	In vitro	VX2	Decrease cell viability in a time-dependent manner, result in cell death [127]
Ovarian carcinoma	In vitro In vivo	ES2, SKOV3, OV90, OVCAR3, CAOV3 ES2 (intraperitoneal injection into female BALB/C mice)	Inhibit cancer cell growth and viability, induce cancer cell apoptosis, inhibit the intraperitoneal metastasis of cancer cells, improve the survival rates of cancer mouse [128]
Melanoma cancer	In vitro In vivo	B16F10 B16F10 (subcutaneous injection into C57BL/6 mice)	Cause cancer cell death, induce immunogenic cancer cell death, reduce tumor mass, increase leukocyte tumor infiltrates in vivo [129]
Breast cancer	In vivo	MCF7, AMJ13, AMN3, HBL AMN3 (intraperitoneal injection into female albino Swiss mice)	Reduce tumor volume, inhibit tumor growth, improve mice weight [130]

#### 4.2.4. NTP Improves the Performance of Biomedical Materials

Biomedical materials are a class of natural or synthetic materials used for disease diagnosis and treatment, repair and replacement of damaged tissues, and enhancement of biological function. The modern field of biomaterials has grown significantly in the past decade due to discoveries in tissue engineering and regenerative medicine. Plasma exposure on biomedical materials can achieve the effects of sterilization and enhancement of histocompatibility, adhesion, and affinity, which provide a good basis for artificial organ transplantation. Ercan et al. [148] found that NTP-produced ROS could effectively kill bacteria such as *S. aureus* and *E. coli* that cause the contamination of sutures without altering their mechanical properties; this is beneficial for preventing infection at the surgical site. In addition to direct damage to biological macromolecules, NTP has an anti-biofilm effect against ESKAPE bacteria in the planktonic and biofilm states, this dual bactericidal effect allows NTP to be used for the sterilization of different types of medical materials [23]. 

NTP-produced reactive species (such as ROS) transform metal ions in liquids into metal nanoparticles, which attach or penetrate into biomaterials, followed by changing the surface properties of biomaterials (roughness and hydrophilicity, etc.), resulting in improvements of cell adhesion and affinity to those materials [27]. The properties of NTP-treated biomaterials are better than the traditional chemical synthesis method because of lower toxicity. This makes NTP more suitable for application in the field of biomedical [27]. Poly(lactide-co-glycolide) (PLGA) particles can be used for the sustained delivery of proteins in the repair process of the nervous system. Coleman et al. [149] used NTP-treated PBS for the sterilization of protein-loaded PLGA particles, the result showed that NTP-treated PBS not only decontaminated the PLGA particles, but also maintained the activity of proteins effectively. Moreover, the efficacy of NTP in enhancing the histocompatibility of certain biomedical materials was dependent on the levels of NTP-generated ROS. It has been shown that NTP treatment can enhance the water permeability of interconnected porous calcium hydroxyapatite (IP-CHA) porous structures and the in vivo bone conductivity of IP-CHA discs, thereby improving the efficacy of IP-CHA as a bone substitute [150]. Wang et al. [151] found that NTP can not only be applied to sterilize poly(ether ether ketone) (PEEK), but also be applied to change the surface properties of implantable PEEK through increasing ROS levels [152], thereby improving the proliferation and adhesion of osteoblasts and fibroblasts. NTP technology has been increasingly used in biomedical materials, and the optimization of NTP treatment conditions and more studies in vivo will benefit its clinical application.

## 5. Conclusions

In recent years, NTP has shown great potential for application in many fields. NTP with different treatment conditions can directly or indirectly mediate the levels of ROS. Low or physiological levels of ROS mediated by appropriate NTP treatment promote the proliferation and differentiation of cells and the growth rate and reproductive capacity of livestock and poultry, which can be applied in the fields of tissue regeneration and animal husbandry. In contrast, high or excessive levels of ROS mediated by high-intensity or prolonged NTP treatment cause oxidative stress damage and death of cells or microorganisms, which can be used in many fields such as environmental disinfection, food production and storage, wound healing, oral treatment, cancer therapy, and biomedical materials. The desired effects of NTP treatment on biological targets can be achieved mainly based on the optimization of its parameters, which can mediate ROS levels through three aspects (production of exogenous ROS, stimulatory generation of intracellular ROS, and regulation of intracellular ROS). However, the concentration and lifetime of exogenous ROS produced by NTP, the penetration and distance of NTP-produced exogenous ROS delivered into cells and how to generate intracellular ROS, the mechanism of how NTP interacts with various cellular components to stimulate generation of intracellular ROS, and the mechanism of how NTP affects redox balance-related transcription factors and signaling pathways to regulate intracellular ROS levels, etc., need to be well clarified for comprehensive understanding the mechanisms of NTP-mediated ROS and accurately regulating the effectiveness of its applications. In addition, the exact radical or reactive components produced by different types of NTP devices, the accurate concentrations and lifetimes of those components, and the interaction between NTP and biological targets need to be well investigated for the development of NTP technology. New directions for the future are provided by the innovative combination of NTP with other strategies and the creation of novel NTP-responsive devices for various fields, which will be conducive to promoting the healthy development of animal husbandry and the prevention and treatment of diseases in both animals and human beings. 

## Data Availability

Not applicable.
